# Mass Spectrometry Analysis Reveals Lipids Induced by Oxidative Stress in *Candida albicans* Extracellular Vesicles

**DOI:** 10.3390/microorganisms11071669

**Published:** 2023-06-27

**Authors:** Gabriel Trentin, Tamires A. Bitencourt, Arthur Guedes, André M. Pessoni, Veronica S. Brauer, Alana Kelyene Pereira, Jonas Henrique Costa, Taicia Pacheco Fill, Fausto Almeida

**Affiliations:** 1 Department of Biochemistry and Immunology, Ribeirão Preto Medical School, University of São Paulo, Ribeirão Preto 14049-900, Brazil; 2 Department of Organic Chemistry, Institute of Chemistry, University of Campinas, Campinas 13083-970, Brazil

**Keywords:** *Candida albicans*, extracellular vesicles, virulence factor, metabolomics, biomolecules

## Abstract

*Candida albicans* is a commensal fungus in healthy humans that causes infection in immunocompromised individuals through the secretion of several virulence factors. The successful establishment of infection is owing to elaborate strategies to cope with defensive molecules secreted by the host, including responses toward oxidative stress. Extracellular vesicle (EV) release is considered an alternative to the biomolecule secretory mechanism that favors fungal interactions with the host cells. During candidiasis establishment, the host environment becomes oxidative, and it impacts EV release and cargo. To simulate the host oxidative environment, we added menadione (an oxidative stress inducer) to the culture medium, and we explored *C*. *albicans* EV metabolites by metabolomics analysis. This study characterized lipidic molecules transported to an extracellular milieu by *C. albicans* after menadione exposure. Through Liquid Chromatography coupled with Mass Spectrometry (LC-MS) analyses, we identified biomolecules transported by EVs and supernatant. The identified molecules are related to several biological processes, such as glycerophospholipid and sphingolipid pathways, which may act at different levels by tuning compound production in accordance with cell requirements that favor a myriad of adaptive responses. Taken together, our results provide new insights into the role of EVs in fungal biology and host–pathogen interactions.

## 1. Introduction

Fungal infections are responsible for over 1.6 million deaths per year [1,2]. *Candida* spp. are the main etiologic agents of systemic fungal infections, with *C. albicans* being the most prevalent species [3]. *C. albicans* is a commensal and dimorphic fungus that may cause infection in immunocompromised individuals [4,5].

*C. albicans* infection is characterized by morphogenetic transitions, in which saprophytic fungal cells convert themselves to parasitic hyphae [4,6]. This transition process, associated with the employment of several virulence factors, allows the establishment of *C. albicans* in the host and the development of candidiasis. As an immune defense response to invasion, phagocytes attack several reactive chemicals in an attempt to kill invading microorganisms [7,8]. The production of Reactive Oxygen Species (ROS) by phagocytes results in oxidative stress, stimulating a respiratory burst [8,9]. In host–pathogen interactions and as an adaptive response, *C. albicans* employs biomolecules and metabolic pathways to attenuate oxidative damage [10,11].

Biomolecule secretory mechanisms are associated with virulence events that lead to the breakdown of physical barriers, host cell adhesion, and immune defense evasion. Taken together, these events allow for the establishment of an infection [2,6,12,13]. Identification of these virulence-associated molecules is a key to understanding pathogenic processes and establishing strategies to design new drugs [2,6].

Extracellular vesicles (EVs) are spherical bilayered compartments secreted by all live cells [12,14]. EV production was reported in several fungal species, such as *Aspergillus flavus* [15], *Aspergillus fumigatus* [16], *C. albicans* [12], *Candida parapsilosis* [12], *Cryptococcus gattii* [17], *Histoplasma capsulatum* [12], *Malassezia sympodialis* [18], *Sporothrix brasiliensis* [19], *Sporothrix schenckii* [12], *Saccharomyces cerevisiae* [20], *Paracoccidioides brasiliensis* [21], *Trichophyton interdigitale* [22], and several other fungi. EVs are composed of proteins, polysaccharides, lipids, RNA, and pigments, and these structures may be associated with pathogenesis during fungal infection [6,12,13,23,24,25]. The *C*. *albicans* EVs play an essential role during candidiasis establishment, and the virulence-associated molecules have been identified [6,12,24,26]. These systematic characterizations allow for a better understanding of fungal physiopathology [15]. However, the molecules induced by oxidative stress have not yet been characterized.

During oxidative stress, the ROS overproduction triggers damage to several cellular components and process [9]. Specifically, lipids are susceptible to lipids peroxidation by ROS attack, leading to structural modifications, which affects membrane integrity [27,28]. Since the EVs may be plasma-membrane-derived, and lipids play a crucial role in EV biogenesis [29,30], any plasma membrane injury may alter EV production. EV cargo and production reflects the cell state [31]. The oxidative stress response alters the fungal cells’ metabolism [9] and may also affect the EVs’ release and content [32]. The impact of oxidative stress over *Candida albicans* extracellular vesicles is still poorly understood. This study reports the presence of metabolites in *C. albicans* EVs after menadione exposure. We characterized the metabolites using Liquid Chromatography coupled with Mass Spectrometry (LC-MS) analyses. The identified molecules may be a component of EVs’ membrane and associated with oxidative stress response.

## 2. Materials and Methods

### 2.1. Growth Conditions

*C. albicans* strain ATCC 64548 was used in all experiments performed in this study. Yeast cells were cultivated in Sabouraud solid medium (Oxoid, Basingstoke, UK) for 72 h at 30 °C, as previously described [6].

### 2.2. Susceptibility Test

The susceptibility of *C. albicans* strain ATCC 64548 towards menadione was evaluated by the determination of Minimum Inhibitory Concentrations (MIC), as described by the Clinical Laboratory Standard Institute [33]. Menadione (Sigma-Aldrich, St. Louis, MO, USA) was tested in the range of 2500 uM to 4.8 uM. The microtiter plates were incubated for 48 h at 30 °C. Then, MIC_100_ was determined as the lowest concentration that inhibited the growth of *C. albicans*. The assay was performed in three biological replicates. Then, we determined the minimal fungicidal concentration (MFC) by inoculating the MIC_100_ concentration and two higher concentrations on Sabouraud plates at 30 °C for 48 h.

### 2.3. Extracellular Vesicle Isolation, Characterization, and Quantification

EVs were isolated as previously described [34] with slight modifications. We compared the two conditions (control and oxidative stress conditions). Under the oxidative stress condition, we added 46.8 μM of menadione to 20 mL Sabouraud solid medium. Sabouraud medium alone was used as the control. Ten isolated colonies were inoculated in 10 mL Sabouraud dextrose liquid medium (Oxoid, Basingstoke, UK) and cultivated under agitation (200 rpm) at 30 °C for 48 h. The fungal cells were counted and calculated to a final concentration of 3.5 × 10^6^ cells/mL, and they were spread onto Sabouraud solid medium and incubated for 24 h at 30 °C [34]. Yeast cells were harvested from solid medium and transferred to a centrifuge tube containing 30 mL of sterile phosphate-buffered saline. Fungal cells were separated from the supernatant using centrifugation at 5000× *g* for 15 min. The supernatant was harvested and centrifuged at 15,000× *g* for 15 min. The resulting supernatant was concentrated using an Amicon ultrafiltration system (100 kDa cutoff). The liquid (EV-free) from the ultrafiltration system was collected and stored at 4 °C. The concentrate was then centrifuged again at 15,000× *g* for 15 min. The EV suspension was ultracentrifuged at 100,000× *g* for 60 min. The pellet (only EVs) was resuspended in 1 mL sterile nuclease-free water (Sigma Aldrich). All steps were conducted at 4 °C. Characterization and quantification of EV preparation was performed using nanoparticle tracking analysis (NTA) in NanoSight NS300 (Malvern Instruments, Malvern, UK), as previously described [22]. The experiments were performed in triplicate.

### 2.4. Liquid Chromatography–Mass Spectrometry (LC-MS) Analyses

The metabolites in the EV and supernatant samples were extracted with methanol in an ultrasonic bath for 40 min and further dried under inert conditions, as previously described [35]. The dried extracts were diluted in 100 µL methanol, filtered, and loaded in a mass spectrometer. UHPLC-MS/MS analyses were performed using a Thermo Scientific QExactive^®^hybrid Quadrupole-Orbitrap mass spectrometer. The chromatographic conditions were as follows: as the stationary phase, we used a Thermo Scientific Accucore C18 2.6 µm (2.1 mm × 100 mm) column. Mobile phases were 0.1% (*v*/*v*) formic acid in water (A) and acetonitrile (B). Eluent profile (A:B) 0–10 min, gradient from 95:5 up to 2:98; held for 5 min; 15–16.2 min gradient up to 95:5; held for 8.8 min. The flow rate was 0.2 mL min^−1^ with an injection volume of 3 µL. For the Mass Spectrometry analysis, we used electrospray ionization in positive mode, the capillary voltage at +3.5 kV; the capillary temperature at 250 °C; S-lens of 50 V and *m*/*z* range of 133.40–2000.00. MS/MS was performed using normalized collision energy (NCE) of 30 eV, and 5 precursors per cycle were selected. Stationary phase: operation and spectra analysis were conducted using Xcalibur software (version 3.0.63) developed by Thermo Fisher Scientific (Waltham, MA, USA).

### 2.5. Molecular Networking and Metabolomic Analyses

The metadata and data from Xcalibur software were converted to .mzML using the MSConvert software (http://proteowizard.sourceforge.net (accessed on 1 October 2020). A molecular network for *C. albicans* (EVs and supernatant metabolites) was created using the online workflow (https://ccms-ucsd.github.io/GNPSDocumentation/networking/ (accessed on 1 October 2020) on the Global Natural Products Social (GNPS) molecular networking website platform (http://gnps.ucsd.edu) using the Classical Molecular Networking (CMN) tool. For CMN, the data were filtered by removing all MS/MS fragment ions within ±17 Da of precursor *m*/*z*. MS/MS spectra were window-filtered by choosing only the top six fragment ions in the ±50 Da window throughout the spectrum. The precursor ion mass tolerance was set to 0.02 Da and an MS/MS fragment ion tolerance of 0.02 Da. A network was then created where edges were filtered to have a cosine score above 0.5 and more than five matched peaks. Furthermore, the edges between two nodes were retained in the network, if and only if each of the nodes appeared in each of the top 10 most similar nodes. Finally, the maximum size of a molecular family was set to 100, and the lowest scoring edges were removed from the molecular families until the molecular family size was below this threshold. The spectra in the network were then searched against the GNPS spectral libraries. The library spectra were filtered in the same manner as for the input data. All matches kept between network spectra and library spectra were required to have a score above 0.5 and at least 5 matched peaks [36]. The resulting molecular network is available at https://gnps.ucsd.edu/ProteoSAFe/status.jsp?task=92af3c25e2d147c5af0e619b52bb0667 (accessed on 1 October 2020). Other studies using Network Annotation Propagation (NAP) and MolNetEnhancer tools were carried out on the GNPS platform. The nodes (compounds) originating from Sabouraud media and solvent analyses (methanol) were excluded from the original network to enable visualization of metabolites derived from control and oxidative stress conditions [37]. Finally, the final spectral network (.cys) was uploaded to Cytoscape 3.8 to obtain better visualization and editing. To improve the data visualization, GNPS Dashboard was used in ‘feature finding’ process using MZMine 2. The parameters used were precursor tolerance: 10 ppm; noise level: 10 × 10^4^; minimum and maximum peak width: 0.05–1.5 min; and retention time tolerance: 0.3 min. The quantification table data were submitted to MetaboAnalyst 5.0 (https://www.metaboanalyst.ca/ (accessed on 1 October 2020) to enhance univariate and multivariate statistical analyses. Semiquantitative evaluations were performed based on Feature-Based Molecular Networking (FBMN), according to the GNPS workflow (https://ccms-ucsd.github.io/GNPSDocumentation/feature-based/molecular/networking/ (accessed on 1 October 2020).

## 3. Results

### 3.1. C. albicans Extracellular Vesicle Profile

During the infection process, host cells use high levels of oxidative chemical compounds to counteract fungal invasion. To mimic the oxidative stress environment in the host milieu, we added menadione to Sabouraud medium (an oxidative stress condition) and compared the EV metabolite content in this condition with the EV metabolite content in Sabouraud medium (control). We inoculated the MIC_100_ (156 μM) in Sabouraud plates and observed insignificant growth. We needed an expressive growth to isolate EVs, and for that reason, we used MIC_30_ (46.8 μM).

**Figure 1:**
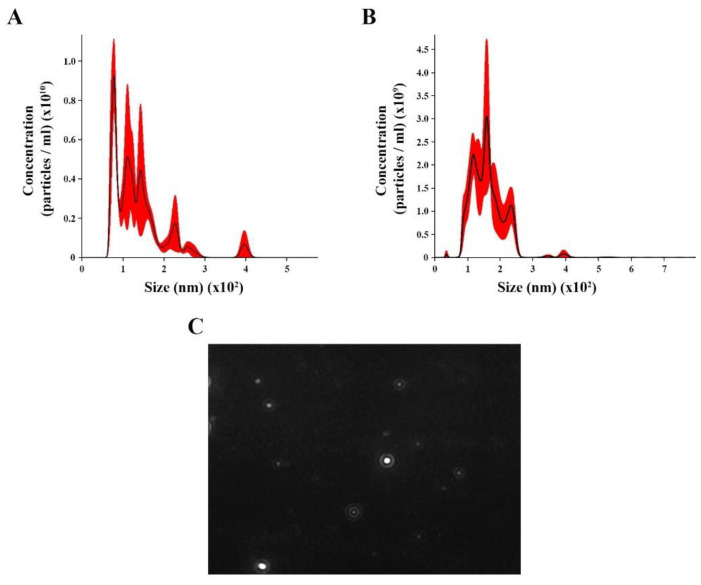
Nanoparticle tracking analysis (NTA) of extracellular vesicles (EVs) released by *Candida albicans*: EVs were isolated from *C. albicans* solid media analyzed through NTA. (**A**) The histogram showing *C. albicans* EV profile distribution (EVs × 10^10^/mL versus size distribution (nm)) in control. (**B**) The histogram showing *Candida albicans* EV profiles distribution (EVs × 10^11^/mL versus size distribution (nm)) in stress oxidative condition. (**C**) Screenshot obtained from a video recording generated in Nanosight NS300 showing EVs from *C. albicans*.

We isolated and concentrated EVs from *C. albicans* yeast cells under control (with no treatment) and oxidative stress conditions from *C. albicans* yeast cells. NTA analysis for control revealed EV heterogeneous size ranging from 70 to 400 nm (Figure 1A), with an average size of 136.4 nm (±58.5 nm) and mode diameter of approximately 100.3 nm. The EVs under oxidative stress showed a different range size (36 to 294 nm), with an average of 160.6 nm (±51.6 nm) and a mode diameter of approximately 158.7 (Figure 1B). A screenshot of *C. albicans* EVs was obtained from a video recording generated using the Nanosight NS300 system (Figure 1C).

### 3.2. Statistical and Molecular Networking Analyses in C. albicans EVs

**Figure 2:**
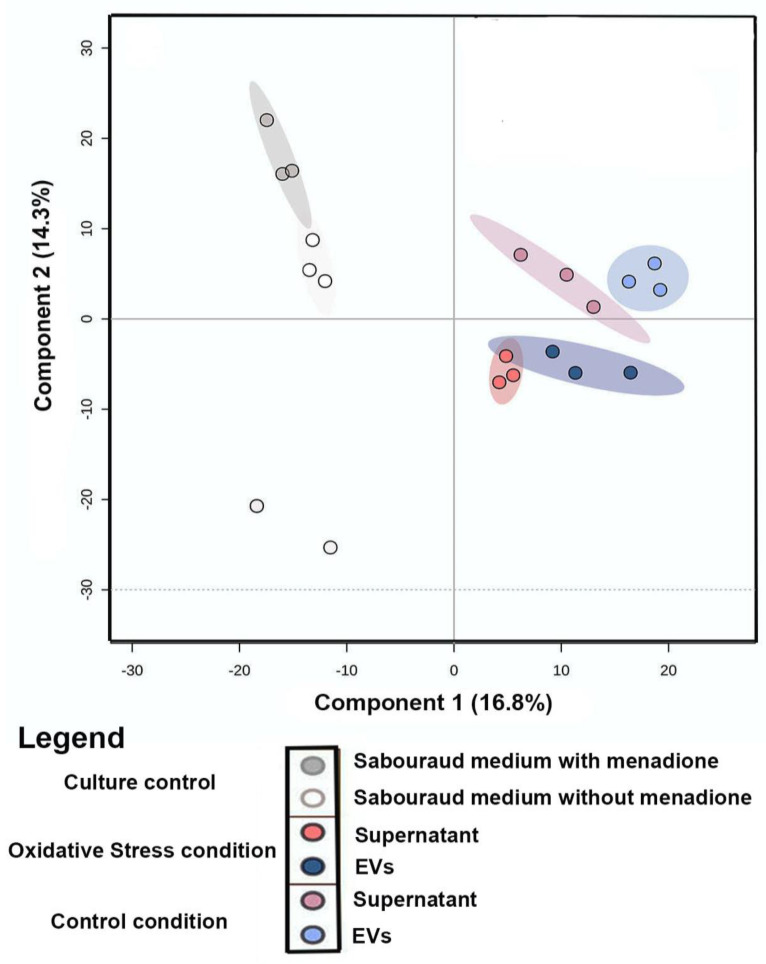
Statistical Analysis of LC−MS/MS data of the *C. albicans* EVs in oxidative stress and control conditions: Partial Least Square (Discriminant Analysis) (PLS-DA) of samples (EVs and supernatant) from control/oxidative stress and culture medium control.

Principal Component Analysis (PCA) of the comparison between metabolites produced under oxidative stress (EVs and supernatant) and control conditions (EVs, supernatant, and culture media) was performed to evaluate the grouping tendencies. According to the PCA, the samples did not form a distinct cluster. Both principal components (PC1 and PC2) were responsible for 33.7% of the total data variance and did not show a clear separation between the main groups of control and samples under oxidative stress. For this reason, Partial Least Square (discriminant analysis) (PLS-DA) (Figure 2) was performed. The PLS-DA principal components were responsible for 31.11% of the total data variance (16.8% for PC1 and 14.3% for PC2) and exhibited a clear separation between the conditions evaluated. Discriminant analyses revealed validated parameters (*p* < 0.01, R2 = 0.99, and Q2 = 0.71). The culture media control was performed to remove Sabouraud medium interferents and show the particular behavior of culture control clusters. To analyze the chemical composition (EVs and supernatant) under control and stress conditions, a classic molecular network based on MS data was generated using the GNPS platform [37]. In both conditions, Molecular Networking consisted of 2940 nodes, in which each node indicated an MS spectrum. Some chemical families detected included sphingolipids and glycerophospholipids, which were annotated by the spectral library. Other chemical superclasses were also annotated, e.g., fatty acyls, steroids, benzene, and derivatives.

### 3.3. Metabolites Annotation

During *C. albicans* infection, the fungus copes with defensive mechanisms triggered by host cells, such as oxidative stress caused by phagocytes [9]. From the menadione MIC_100_ determination, we set the MIC_30_ concentration to be used for EV isolation to 46.8 μM of menadione. During the isolation process, we hypothesized the occurrence of canonical (transporter) and noncanonical (via EVs) metabolite secretions. For this reason, filtered EVs were collected to investigate the possible presence of metabolites. To rule out any interference from Sabouraud medium, the ions present in the control were subtracted during LC-MS/MS analysis.

**Figure 3:**
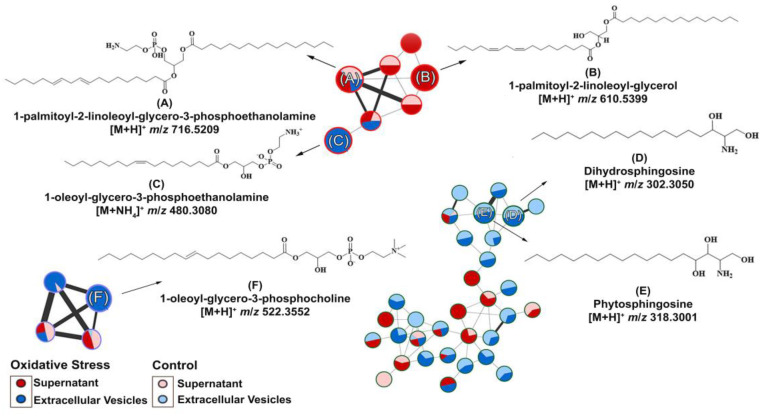
Molecular network and chemical structure obtained for *Candida albicans* supernatant/extracellular vesicles through LC-MS/MS analysis: A GNPS molecular network of six metabolites were identified. The GNPS grouped the metabolites according to their structural functions, percentage in supernatant or EVs, and their similarity. The glycerophospholipid compounds were grouped in clusters (**A**–**C**,**F**), whereas the sphingolipids were grouped in clusters (**D**,**E**).

As we isolated *C. albicans* EVs under both conditions, we aimed to identify whether metabolite content could be differentially produced by oxidative stress stimuli. Therefore, metabolites were extracted from these EVs under both conditions and compared. EV isolation yielded approximately 10^10^ EVs/mL (control) and 10^11^ EVs/mL (oxidative stress conditions). The different yields obtained among the analyzed conditions were expected because of the reduced yeast growth upon menadione exposure. We predefined MIC_30_ concentration of menadione exposure as a concentration to promote stress and, in the meanwhile, to allow yeast growth enough for EV extraction for downstream application. The purified EVs were further extracted, and their chemical composition was analyzed by Liquid Chromatography–Mass Spectrometry (Figure S1). The data obtained were submitted to the GNPS platform for metabolite annotation. Metabolite fragmentation profiles were compared and annotated as hits in the GNPS database (Supplementary material). Six metabolites were annotated in the network (Figure 3). The nodes represent each chemical and the lines the similarity between them.

Compounds **A**–**F** (Table 1) were divided according to their metabolic functions: glycerophospholipid metabolic pathways and sphingolipid biosynthesis. Compounds (**A**) (Figure 3A) and (**B**) (Figure 3B) were identified in the supernatants of EV isolation. Regarding compounds related to glycerophospholipid metabolism identified in the supernatant, we detected the precursor ion [M + H]^+^ *m*/*z* 716.5220, annotated as 1-palmitoyl-2-linoleoyl-glycero-3-phosphoethanolamine (**A**), present in control and oxidative stress conditions, which yielded the main fragments at *m*/*z* 575.5100, characteristic of phosphoethanolamine moiety loss, and *m*/*z* 95.0860, both matching the spectral library on GNPS (Figure S2) [38]. Compound 1-palmitoyl-2-linoleoyl-glycerol (**B**) was induced under oxidative stress conditions and annotated with the precursor ion [M + NH_4_]^+^ at *m*/*z* 610.5400. Most of the main fragments of compound (**B**) matched the fragmentation patterns found in the GNPS database (Figure S3). The product ions at *m*/*z* 313.27 and 337.27 correspond to the loss of palmitic and linoleic acid [39], respectively.

In EVs under oxidative stress conditions, compound (**C**) was induced, and the precursor ion [M + H]^+^ at *m*/*z* 480.3080 was annotated with high similarity to the spectral library (Figure S4) of the metabolite 1-oleoyl-glycero-3-phosphoethanolamine (Figure 3C). The ion at *m*/*z* 339.2910 is a major fragment that corresponds to dehydrated oleoyl (18:1) glycerol, which was predicted by the HMDB database and previously described [40].

In both conditions, compounds (**D**) (Figure 3D) and (**E**) (Figure 3E) were identified in EVs. Compound dihydrosphingosine (**D**) with a precursor ion at *m*/*z* [M + H]^+^ 302.3050. (**D**) shows the ions at *m*/*z* 254.2850, 95.0860, 81.0710, and 60.0450 as the main fragments [41]. The loss of the moiety [H_2_NCHCH_2_OH]^+^ is known for compound (**D**) as the ion at *m*/*z* 60.0450 and the base peak according to the HMDB spectral and GNPS database (Figure S5). In addition, the ion [M + H]^+^ at *m*/*z* 318.3000 was annotated in the GNPS database (Figure S6) as phytosphingosine (**E**), and the MS/MS experiment yielded major fragments at *m*/*z* 282.2780 and 60.0450, which corresponded to di-dehydration and to the fragment [H_2_NCHCH_2_OH]^+^, as expected for compound (**E**) and experimentally found in the HMDB database [42].

The metabolite 1-oleoyl-glycero-3-phosphocholine (**F**) (Figure 3F) was also induced in EVs during oxidative stress and was annotated with the precursor ion [M + H]^+^ at *m*/*z* 522.350. MS/MS indicated the main fragments at *m*/*z* 86.10, 184.0730, and 104.1080, corresponding to choline fragmentation, phosphocholine, and choline ions, respectively [43]. These fragments were related to the amino-phosphate moiety loss and the cleavage between the oxygen–phosphorus bond, respectively, as can be seen in the spectral library in GNPS (Figure S7) and predicted in the HMDB spectral database. Spectral matches are available in the Supplementary Materials.

## 4. Discussion

Previously, we described the role of EVs during yeast-to-hypha transition and fungal cell communication in *C. albicans* strain (ATCC 64548) [44]. Zang et al. measured ROS production in the same strain [45]. Herein, we demonstrated that EVs from *C. albicans* present different metabolite contents, according to environmental changes. The oxidative stress condition was designed to simulate one of the events that occurs during the establishment of *C. albicans* infection. These changes were triggered by menadione addition to mimic the overproduction of ROS caused by a phagocytic milieu [46]. Menadione (or vitamin K_3_) is a quinone class compound [47,48]. This compound has been used as a model to study damage related to oxidative stress [47,48]. Menadione induced by the semiquinone redox process stimulates ROS production [47,48]. High levels of ROS alter cellular redox homeostasis [49]. Moreover, oxygen forms are highly harmful to cellular integrity, affecting cell growth and physiological functions [46,50]. The yields of EVs were greater for *Candida albicans* cells exposed to menadione (10^11^ EVs/mL) than control condition (10^10^ EVs/mL). We attribute this behavior to yeast cell response to oxidative stress. The stress response may regulate the vesicle traffic [51]. In this respect, we compared the EV metabolite content under oxidative conditions with the EV metabolite content in the control condition (a nonstress stimuli condition). We also collected the supernatant and evaluated the possible presence of metabolites regardless of the EV loading molecules. EV content is regulated by oxidative stress.

**Figure 4:**
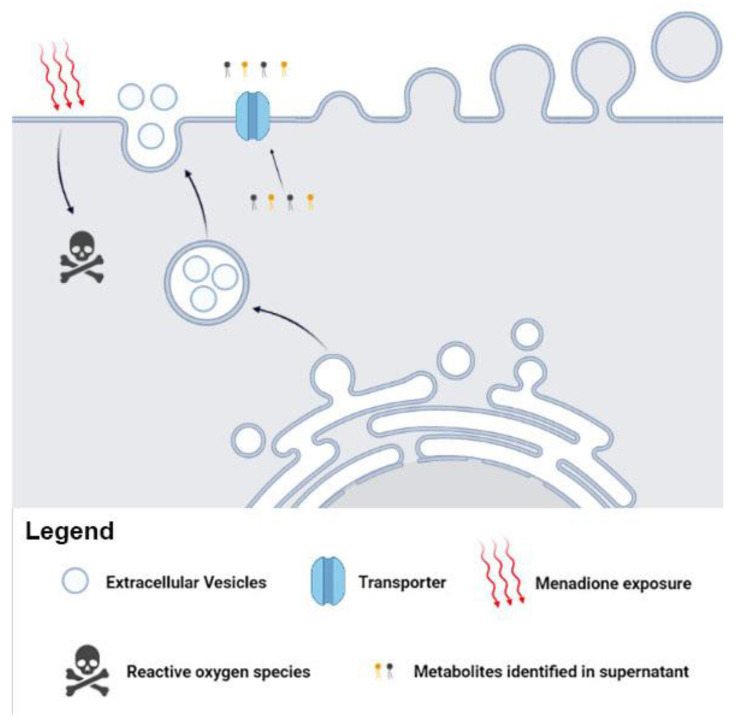
Metabolites can influence fungal mechanisms and alter the infection process. The *Candida albicans* EVs are involved in fungal pathogenesis and transport specific metabolites after menadione exposure. These metabolites may influence biological processes and affect several fungal adaptive responses.

We suggest the possible metabolites release via canonical (transporter), noncanonical (EVs), or both pathways in *C. albicans*. The presence of metabolites in supernatant and in the EVs supports our hypothesis of canonical/noncanonical transport (Figure 4). The traffic of fungal biomolecules is coordinated in response to environmental changes [52,53]. In addition, communication mediated by *C. albicans* EVs has been reported, demonstrating the vital role of EVs in environmental stress.

The presence of 1-palmitoyl-2-linoleoyl-glycero-3-phosphoethanolamine in the supernatant and EVs under both conditions suggests that the release of these molecules is independent of cell redox homeostasis. On the other hand, the high amount of ROS leads to lipid peroxidation by a free radical chain reaction and may cause plasma membrane injure, affecting its physical propriety and dynamism [9,32]. Some membrane polyunsaturated fatty acids are the most susceptible to ROS attack [32]. Biomolecules react to ROS exposure, and previous studies have reported the release of metabolites by fungi during oxidative stress [54,55,56]. Furthermore, some fungal species try to avoid ROS attack through glycerophospholipid hydrolysis [57], which may justify the presence of compounds 1-palmitoyl-2-linoleoyl-glycerol, 1-oleoyl-glycero-3-phosphoethanolamine, and 1-oleoyl-glycero-3-phosphocholine in the supernatant and EVs from the menadione culture. These glycerophospholipids are likely to be involved in oxidative stress pathways. Under oxidative conditions, glycerophospholipids undergo structural modifications that affect the membrane integrity [58]. In addition, our data corroborate the occurrence of metabolite transport in other vias, as previously shown in *A. fumigatus* [59].

In yeasts, sphingolipid biosynthesis initiates with an enzymatic conjugation of serine and fatty acyl-CoA, yielding 3-ketodihydrosphingosine [60]. The next step produces dihydrosphingosine (or sphinganine) dihydrosphingosine, which may be converted to phytosphingosine through hydroxylation at carbon 4 [61]. The same mass fragment pattern obtained in our data was previously described for dihydrosphingosine and phytosphingosine in analyses of pathogenic strains of *Candida albicans* and *Cryptococcus* spp. [62,63]. Previous studies also evaluated the metabolome of *C. albicans* under different growth conditions or exposed to antifungals and identified similar classes of metabolites, such as glycerophospholipid metabolism and sphingolipid metabolism [62,64,65].

The compounds 1-oleoyl-glycero-3-phosphoethanolamine (Lyso-PE [18:1]) 1-oleoyl-glycero-3-phosphoethanolamine and 1-oleoyl-glycero-3-phosphocholine (Lyso-PC [18:1]) may also be related to glycerophospholipid pathways [66]. These compounds are classified as lysophosphatidylethanolamines (LysoPE) and lysophosphatidylcholines (LysoPC), respectively, and the same mass fragment pattern obtained in our data was previously described for 1-oleoyl-glycero-3-phosphoethanolamine and 1-oleoyl-glycero-3-phosphocholine [43]. Intermediates of these pathways are the building blocks for most subcellular membranes [67]. A recent study identified LysoPE and LysoPC compounds in the pathogenic fungal *Histoplasma capsulatum* [68]. In addition, the characterization of glycerophospholipids and phospholipids in EV content has been described for the pathogenic phase of *Paracoccidioides brasiliensis*, *C. albicans*, and *Candida auris* [69,70]. The induction of these metabolites during oxidative stress in EVs may reflect an imbalance in glycerophospholipid and phospholipid homeostasis caused by high ROS levels [71,72]. Furthermore, some lysophospholipids are directly linked to leukocyte activity and may act as virulence factors during infection [69,73,74]. A previous study reported that the integrity of fungal EVs is crucial for transmitting virulence [17]. To ensure cell-to-cell information delivery, EVs may be internalized by the acceptor cell; however, these mechanisms are not completely characterized [44,75]. The exclusive presence of LysoPC and LysoPE compounds in *C. albicans* EVs reflected the response to oxidative stress caused by menadione, and *C. albicans* cells may take up these EVs, transmitting the oxidative stress information. Accurate lipidomic analysis should be conducted in an attempt to attribute the exact locale of the identified metabolites (EVs membrane or cargo). The identified molecules have their particular pathways’ biosynthesis and may not participate in central metabolism, which is involved in energy generation.

The EVs can assist in environmental sensing, showing alterations in their sorting of molecules in accordance with cellular requirements [14], which was reinforced by our data, highlighting the occurrence of specific metabolites after menadione exposure. Unveiling the metabolites content within *C. albicans* EVs may be useful to generate more information about the mechanisms underlying EV communication that may favor the fungal infection process.

## Figures and Tables

**Table 1:** *C. albicans* metabolites identified and their metabolic pathways.

ID	Compounds	Ion Formula	Experimental *m*/*z*	Retention Time (min)	Supernatant or EVs	Control or Stress Oxidative Condition	Related Pathways
**A**	1-palmitoyl-2-linoleoyl-glycero-3-phosphoethanolamine	C_39_H_74_NO_8_P	716.5209	9.89	Supernatant and EVs	Control and stress oxidative condition	Glycerophospholipid metabolism
**B**	1-palmitoyl-2-linoleoyl-glycerol	C_37_H_68_O_5_	610.5399	15.94	Supernatant	Stress oxidative condition	Glycerophospholipid metabolism
**C**	1-oleoyl-glycero-3-phosphoethanolamine	C_23_H_46_NO_7_P	480.3080	9.41	EVs	Stress oxidative condition	Glycerophospholipid metabolism
**D**	Dihydrosphingosine	C_18_H_39_NO_2_	302.3050	7.94	EVs	Control and stress oxidative condition	Sphingolipids metabolism
**E**	Phytosphingosine	C_18_H_39_NO_3_	318.3001	7.74	EVs	Control and stress oxidative condition	Sphingolipids metabolism
**F**	1-oleoyl-glycero-3-phosphocholine	C_26_H_52_NO_7_P	522.3552	9.74	EVs	Stress oxidative condition	Glycerophospholipid metabolism

## Data Availability

Metabolomics data were deposited in the Global Natural Products Social Molecular Networking (GNPS) database and are available at https://gnps.ucsd.edu/ProteoSAFe/status.jsp?task=92af3c25e2d147c5af0e619b52bb0667 (accessed on 1 October 2022).

## Supplementary Materials

The following supporting information can be downloaded at: https://www.mdpi.com/article/10.3390/microorganisms11071669/s1, Figure S1. Schematic representation of molecular network creation from tandem mass spectra data. Figure S2. GNPS data and extracted ion chromatograms for compound (**A**). Figure S3. GNPS data and extracted ion chromatograms of compound (**B**). Figure S4. GNPS data and extracted ion chromatograms of compound (**C**). Figure S5. GNPS data and extracted ion chromatograms of compound (**D**). Figure S6. GNPS data and extracted ion chromatograms for compound (**E**). Figure S7. GNPS data and extracted ion chromatograms for compound (**F**).

## References

[1] Brown G.D., Denning D.W., Gow N.A., Levitz S.M., Netea M.G., White T.C. (2012). Hidden killers: Human fungal infections. Sci. Transl. Med..

[2] De Toledo Martins S., Szwarc P., Goldenberg S., Alves L.R. (2019). Extracellular Vesicles in Fungi: Composition and Functions. Curr. Top. Microbiol. Immunol..

[3] Almeida F., Rodrigues M.L., Coelho C. (2019). The Still Underestimated Problem of Fungal Diseases Worldwide. Front. Microbiol..

[4] Poulain D. (2015). *Candida albicans*, plasticity and pathogenesis. Crit. Rev. Microbiol..

[5] Schmiedel Y., Zimmerli S. (2016). Common invasive fungal diseases: An overview of invasive candidiasis, aspergillosis, cryptococcosis, and Pneumocystis pneumonia. Swiss Med. Wkly..

[6] Vargas G., Rocha J.D., Oliveira D.L., Albuquerque P.C., Frases S., Santos S.S., Nosanchuk J.D., Gomes A.M., Medeiros L.C., Miranda K. (2015). Compositional and immunobiological analyses of extracellular vesicles released by *Candida albicans*. Cell. Microbiol..

[7] Brown G.D. (2011). Innate Antifungal Immunity: The Key Role of Phagocytes. Annu. Rev. Immunol..

[8] Komalapriya C., Kaloriti D., Tillmann A.T., Yin Z., Herrero-de-Dios C., Jacobsen M.D., Belmonte R.C., Cameron G., Haynes K., Grebogi C. (2015). Integrative Model of Oxidative Stress Adaptation in the Fungal Pathogen *Candida albicans*. PLoS ONE.

[9] Dantas A.d.S., Day A., Ikeh M., Kos I., Achan B., Quinn J. (2015). Oxidative stress responses in the human fungal pathogen, *Candida albicans*. Biomolecules.

[10] Brown A.J.P., Brown G.D., Netea M.G., Gow N.A.R. (2014). Metabolism impacts upon Candida immunogenicity and pathogenicity at multiple levels. Trends Microbiol..

[11] Williams Robert B., Lorenz Michael C., Andrew Alspaugh J. (2020). Multiple Alternative Carbon Pathways Combine to Promote *Candida albicans* Stress Resistance, Immune Interactions, and Virulence. mBio.

[12] Albuquerque P.C., Nakayasu E.S., Rodrigues M.L., Frases S., Casadevall A., Zancope-Oliveira R.M., Almeida I.C., Nosanchuk J.D. (2008). Vesicular transport in Histoplasma capsulatum: An effective mechanism for trans-cell wall transfer of proteins and lipids in ascomycetes. Cell. Microbiol..

[13] Rodrigues M.L., Nimrichter L., Oliveira D.L., Nosanchuk J.D., Casadevall A. (2008). Vesicular Trans-Cell Wall Transport in Fungi: A Mechanism for the Delivery of Virulence-Associated Macromolecules?. Lipid Insights.

[14] Cleare L.G., Zamith D., Heyman H.M., Couvillion S.P., Nimrichter L., Rodrigues M.L., Nakayasu E.S., Nosanchuk J.D. (2020). Media Matters! Alterations in the loading and release of Histoplasma capsulatum extracellular vesicles in response to different nutritional milieus. Cell. Microbiol..

[15] Brauer V.S., Pessoni A.M., Bitencourt T.A., de Paula R.G., de Oliveira Rocha L., Goldman G.H., Almeida F. (2020). Extracellular Vesicles from Aspergillus flavus Induce M1 Polarization In Vitro. mSphere.

[16] Souza J.A.M., Baltazar L.d.M., Carregal V.M., Gouveia-Eufrasio L., de Oliveira A.G., Dias W.G., Campos Rocha M., Rocha de Miranda K., Malavazi I., Santos D.d.A. (2019). Characterization of Aspergillus fumigatus Extracellular Vesicles and Their Effects on Macrophages and Neutrophils Functions. Front. Microbiol..

[17] Bielska E., Sisquella M.A., Aldeieg M., Birch C., O’Donoghue E.J., May R.C. (2018). Pathogen-derived extracellular vesicles mediate virulence in the fatal human pathogen Cryptococcus gattii. Nat. Commun..

[18] Vallhov H., Johansson C., Veerman R.E., Scheynius A. (2020). Extracellular Vesicles Released from the Skin Commensal Yeast Malassezia sympodialis Activate Human Primary Keratinocytes. Front. Cell. Infect. Microbiol..

[19] Ikeda M.A.K., de Almeida J.R.F., Jannuzzi G.P., Cronemberger-Andrade A., Torrecilhas A.C.T., Moretti N.S., da Cunha J.P.C., de Almeida S.R., Ferreira K.S. (2018). Extracellular Vesicles from Sporothrix brasiliensis Are an Important Virulence Factor That Induce an Increase in Fungal Burden in Experimental Sporotrichosis. Front. Microbiol..

[20] Oliveira D.L., Nakayasu E.S., Joffe L.S., Guimaraes A.J., Sobreira T.J., Nosanchuk J.D., Cordero R.J., Frases S., Casadevall A., Almeida I.C. (2010). Characterization of yeast extracellular vesicles: Evidence for the participation of different pathways of cellular traffic in vesicle biogenesis. PLoS ONE.

[21] Vallejo M.C., Matsuo A.L., Ganiko L., Medeiros L.C.S., Miranda K., Silva L.S., Freymüller-Haapalainen E., Sinigaglia-Coimbra R., Almeida I.C., Puccia R. (2011). The Pathogenic Fungus *Paracoccidioides brasiliensis* Exports Extracellular Vesicles Containing Highly Immunogenic α-Galactosyl Epitopes. Eukaryot. Cell..

[22] Bitencourt T.A., Rezende C.P., Quaresemin N.R., Moreno P., Hatanaka O., Rossi A., Martinez-Rossi N.M., Almeida F. (2018). Extracellular Vesicles from the Dermatophyte Trichophyton interdigitale Modulate Macrophage and Keratinocyte Functions. Front. Immunol..

[23] Freitas M.S., Bonato V.L.D., Pessoni A.M., Rodrigues M.L., Casadevall A., Almeida F. (2019). Fungal Extracellular Vesicles as Potential Targets for Immune Interventions. mSphere.

[24] Peres da Silva R., Puccia R., Rodrigues M.L., Oliveira D.L., Joffe L.S., Cesar G.V., Nimrichter L., Goldenberg S., Alves L.R. (2015). Extracellular vesicle-mediated export of fungal RNA. Sci. Rep..

[25] Rodrigues M.L., Nimrichter L., Oliveira D.L., Frases S., Miranda K., Zaragoza O., Alvarez M., Nakouzi A., Feldmesser M., Casadevall A. (2007). Vesicular Polysaccharide Export in *Cryptococcus neoformans* Is a Eukaryotic Solution to the Problem of Fungal Trans-Cell Wall Transport. Eukaryot. Cell..

[26] Gil-Bona A., Llama-Palacios A., Parra C.M., Vivanco F., Nombela C., Monteoliva L., Gil C. (2015). Proteomics unravels extracellular vesicles as carriers of classical cytoplasmic proteins in *Candida albicans*. J. Proteome Res..

[27] Yadav D.K., Kumar S., Choi E.-H., Chaudhary S., Kim M.-H. (2019). Molecular dynamic simulations of oxidized skin lipid bilayer and permeability of reactive oxygen species. Sci. Rep..

[28] Juan C.A., Pérez de la Lastra J.M., Plou F.J., Pérez-Lebeña E. (2021). The Chemistry of Reactive Oxygen Species (ROS) Revisited: Outlining Their Role in Biological Macromolecules (DNA, Lipids and Proteins) and Induced Pathologies. Int. J. Mol. Sci..

[29] Zamith-Miranda D., Peres da Silva R., Couvillion S.P., Bredeweg E.L., Burnet M.C., Coelho C., Camacho E., Nimrichter L., Puccia R., Almeida I.C. (2021). Omics Approaches for Understanding Biogenesis, Composition and Functions of Fungal Extracellular Vesicles. Front. Genet..

[30] Donoso-Quezada J., Ayala-Mar S., González-Valdez J. (2021). The role of lipids in exosome biology and intercellular communication: Function, analytics and applications. Traffic.

[31] Rayamajhi S., Sulthana S., Ferrel C., Shrestha T.B., Aryal S. (2023). Extracellular vesicles production and proteomic cargo varies with incubation time and temperature. Exp. Cell Res..

[32] Chiaradia E., Tancini B., Emiliani C., Delo F., Pellegrino R.M., Tognoloni A., Urbanelli L., Buratta S. (2021). Extracellular Vesicles under Oxidative Stress Conditions: Biological Properties and Physiological Roles. Cells.

[33] CLSI M27-A3 (2008). Reference Method for Broth Dilution Antifungal Susceptibility Testing of Yeasts.

[34] Reis F.C.G., Borges B.S., Jozefowicz L.J., Sena B.A.G., Garcia A.W.A., Medeiros L.C., Martins S.T., Honorato L., Schrank A., Vainstein M.H. (2019). A Novel Protocol for the Isolation of Fungal Extracellular Vesicles Reveals the Participation of a Putative Scramblase in Polysaccharide Export and Capsule Construction in Cryptococcus gattii. mSphere.

[35] Costa J.H., Wassano C.I., Angolini C.F.F., Scherlach K., Hertweck C., Pacheco Fill T. (2019). Antifungal potential of secondary metabolites involved in the interaction between citrus pathogens. Sci. Rep..

[36] Wang M., Carver J.J., Phelan V.V., Sanchez L.M., Garg N., Peng Y., Nguyen D.D., Watrous J., Kapono C.A., Luzzatto-Knaan T. (2016). Sharing and community curation of mass spectrometry data with Global Natural Products Social Molecular Networking. Nat. Biotechnol..

[37] Oppong-Danquah E., Parrot D., Blümel M., Labes A., Tasdemir D. (2018). Molecular Networking-Based Metabolome and Bioactivity Analyses of Marine-Adapted Fungi Co-cultivated With Phytopathogens. Front. Microbiol..

[38] Takahashi R., Fujioka S., Oe T., Lee S.H. (2017). Stable isotope labeling by fatty acids in cell culture (SILFAC) coupled with isotope pattern dependent mass spectrometry for global screening of lipid hydroperoxide-mediated protein modifications. J. Proteom..

[39] Righetti L., Rubert J., Galaverna G., Folloni S., Ranieri R., Stranska-Zachariasova M., Hajslova J., Dall’Asta C. (2016). Characterization and Discrimination of Ancient Grains: A Metabolomics Approach. Int. J. Mol. Sci..

[40] Zhu Z.-J., Schultz A.W., Wang J., Johnson C.H., Yannone S.M., Patti G.J., Siuzdak G. (2013). Liquid chromatography quadrupole time-of-flight mass spectrometry characterization of metabolites guided by the METLIN database. Nat. Protoc..

[41] Ren J., Snider J., Airola M.V., Zhong A., Rana N.A., Obeid L.M., Hannun Y.A. (2018). Quantification of 3-ketodihydrosphingosine using HPLC-ESI-MS/MS to study SPT activity in yeast *Saccharomyces cerevisiae*[S]. J. Lipid Res..

[42] Dapic I., Brkljacic L., Jakasa I., Kobetic R. (2019). Characterization of Ceramides with Phytosphingosine Backbone by Hydrogen-deuterium Exchange Mass Spectrometry. Croat. Chem. Acta.

[43] Suárez-García S., Arola L., Pascual-Serrano A., Arola-Arnal A., Aragonès G., Bladé C., Suárez M. (2017). Development and validation of a UHPLC-ESI-MS/MS method for the simultaneous quantification of mammal lysophosphatidylcholines and lysophosphatidylethanolamines in serum. J. Chromatogr. B..

[44] Bitencourt Tamires A., Hatanaka O., Pessoni Andre M., Freitas Mateus S., Trentin G., Santos P., Rossi A., Martinez-Rossi Nilce M., Alves Lysangela L., Casadevall A. (2022). Fungal Extracellular Vesicles Are Involved in Intraspecies Intracellular Communication. mBio.

[45] Wei L.-H., Yu T., Wang X.-N., Hou J.-X., Wang X., Wang C., Li K.-K., Jing S.-Y., Zhang X. (2019). In Vitro Potent Activity of ɛ-poly-L-lysine against *Candida albicans* and the Underlying Mechanisms. bioRxiv.

[46] Jamieson D.J., Stephen D.W.S., Terrière E.C. (1996). Analysis of the adaptive oxidative stress response of *Candida albicans*. FEMS Microbiol. Lett..

[47] Chiou T.-J., Tzeng W.-F. (2000). The roles of glutathione and antioxidant enzymes in menadione-induced oxidative stress. Toxicology.

[48] Li J., Zuo X., Cheng P., Ren X., Sun S., Xu J., Holmgren A., Lu J. (2019). The production of reactive oxygen species enhanced with the reduction of menadione by active thioredoxin reductase. Metallomics.

[49] Ayer A., Gourlay C.W., Dawes I.W. (2014). Cellular redox homeostasis, reactive oxygen species and replicative ageing in *Saccharomyces cerevisiae*. FEMS Yeast Res..

[50] Jamieson D.J. (1998). Oxidative stress responses of the yeast *Saccharomyces cerevisiae*. Yeast.

[51] Levine A. (2002). Regulation of stress responses by intracellular vesicle trafficking?. Plant. Physiol. Biochem..

[52] Roper M., Dressaire E. (2019). Fungal Biology: Bidirectional Communication across Fungal Networks. Curr. Biol..

[53] Zhou L., Evangelinos M., Wernet V., Eckert Antonia F., Ishitsuka Y., Fischer R., Nienhaus G.U., Takeshita N. (2020). Superresolution and pulse-chase imaging reveal the role of vesicle transport in polar growth of fungal cells. Sci. Adv..

[54] Keller N.P. (2015). Translating biosynthetic gene clusters into fungal armor and weaponry. Nat. Chem. Biol..

[55] Fountain J.C., Bajaj P., Nayak S.N., Yang L., Pandey M.K., Kumar V., Jayale A.S., Chitikineni A., Lee R.D., Kemerait R.C. (2016). Responses of *Aspergillus flavus* to Oxidative Stress Are Related to Fungal Development Regulator, Antioxidant Enzyme, and Secondary Metabolite Biosynthetic Gene Expression. Front. Microbiol..

[56] Martín J.F. (2020). Transport systems, intracellular traffic of intermediates and secretion of β-lactam antibiotics in fungi. Fungal Biol. Biotechnol..

[57] Zhang C., Wang W., Lu R., Jin S., Chen Y., Fan M., Huang B., Li Z., Hu F. (2016). Metabolic responses of Beauveria bassiana to hydrogen peroxide-induced oxidative stress using an LC-MS-based metabolomics approach. J. Invertebr. Pathol..

[58] Domingues M.R.M., Simões C., da Costa J.P., Reis A., Domingues P. (2009). Identification of 1-palmitoyl-2-linoleoyl-phosphatidylethanolamine modifications under oxidative stress conditions by LC-MS/MS. Biomed. Chromatogr..

[59] Wang D.-N., Toyotome T., Muraosa Y., Watanabe A., Wuren T., Bunsupa S., Aoyagi K., Yamazaki M., Takino M., Kamei K. (2014). GliA in Aspergillus fumigatus is required for its tolerance to gliotoxin and affects the amount of extracellular and intracellular gliotoxin. Med. Mycol..

[60] Klug L., Daum G. (2014). Yeast lipid metabolism at a glance. FEMS Yeast Res..

[61] Ren J., Hannun Y. (2019). Metabolism and Roles of Sphingolipids in Yeast Saccharomyces cerevisiae.

[62] Li L., Liao Z., Yang Y., Lv L., Cao Y., Zhu Z. (2018). Metabolomic profiling for the identification of potential biomarkers involved in a laboratory azole resistance in *Candida albicans*. PLoS ONE.

[63] Singh A., MacKenzie A., Girnun G., Del Poeta M. (2017). Analysis of sphingolipids, sterols, and phospholipids in human pathogenic Cryptococcus strains. J. Lipid Res..

[64] Pan J., Hu C., Yu J.-H. (2018). Lipid Biosynthesis as an Antifungal Target. J. Fungi.

[65] Sant D.G., Tupe S.G., Ramana C.V., Deshpande M.V. (2016). Fungal cell membrane—Promising drug target for antifungal therapy. J. Appl. Microbiol..

[66] Rattray J.B., Schibeci A., Kidby D.K. (1975). Lipids of yeasts. Bacteriol. Rev..

[67] Henry S.A., Kohlwein S.D., Carman G.M. (2012). Metabolism and Regulation of Glycerolipids in the Yeast *Saccharomyces cerevisiae*. Genetics.

[68] Zamith-Miranda D., Heyman Heino M., Burnet Meagan C., Couvillion Sneha P., Zheng X., Munoz N., Nelson William C., Kyle Jennifer E., Zink Erika M., Weitz Karl K. (2021). A Histoplasma capsulatum Lipid Metabolic Map Identifies Antifungal Targets. mBio.

[69] Zamith-Miranda D., Heyman Heino M., Couvillion Sneha P., Cordero Radames J.B., Rodrigues Marcio L., Nimrichter L., Casadevall A., Amatuzzi Rafaela F., Alves Lysangela R., Nakayasu Ernesto S. (2021). Comparative Molecular and Immunoregulatory Analysis of Extracellular Vesicles from *Candida albicans* and *Candida auris*. mSystems.

[70] Vallejo M.C., Nakayasu E.S., Longo L.V.G., Ganiko L., Lopes F.G., Matsuo A.L., Almeida I.C., Puccia R. (2012). Lipidomic Analysis of Extracellular Vesicles from the Pathogenic Phase of *Paracoccidioides brasiliensis*. PLoS ONE.

[71] Wang J., Wang H., Zhang C., Wu T., Ma Z., Chen Y. (2019). Phospholipid homeostasis plays an important role in fungal development, fungicide resistance and virulence in *Fusarium graminearum*. Phytopathol. Res..

[72] da Silveira dos Santos A.X., Riezman I., Aguilera-Romero M.-A., David F., Piccolis M., Loewith R., Schaad O., Riezman H. (2014). Systematic lipidomic analysis of yeast protein kinase and phosphatase mutants reveals novel insights into regulation of lipid homeostasis. Mol. Biol. Cell.

[73] Silva-Neto M.A.C., Lopes A.H., Atella G.C. (2016). Here, There, and Everywhere: The Ubiquitous Distribution of the Immunosignaling Molecule Lysophosphatidylcholine and Its Role on Chagas Disease. Front. Immunol..

[74] Tounsi N., Meghari S., Moser M., Djerdjouri B. (2015). Lysophosphatidylcholine exacerbates *Leishmania major*-dendritic cell infection through interleukin-10 and a burst in arginase1 and indoleamine 2,3-dioxygenase activities. Int. Immunopharmacol..

[75] Mathieu M., Martin-Jaular L., Lavieu G., Théry C. (2019). Specificities of secretion and uptake of exosomes and other extracellular vesicles for cell-to-cell communication. Nat. Cell. Biol..

