# Supplementary material for: Mass Spectrometry Analysis Reveals Lipids Induced by Oxidative Stress in Candida albicans Extracellular Vesicles
Source: Microorganisms. 2023 Jun 27;11(7):1669. doi: 10.3390/microorganisms11071669 (PMC10383470; doi:10.3390/microorganisms11071669)
Supplement: Supplementary file 1 [file microorganisms-11-01669-s001.zip › microorganisms-2396878-supplementary.pdf]

microorganisms
MDPI

Supplementary Material

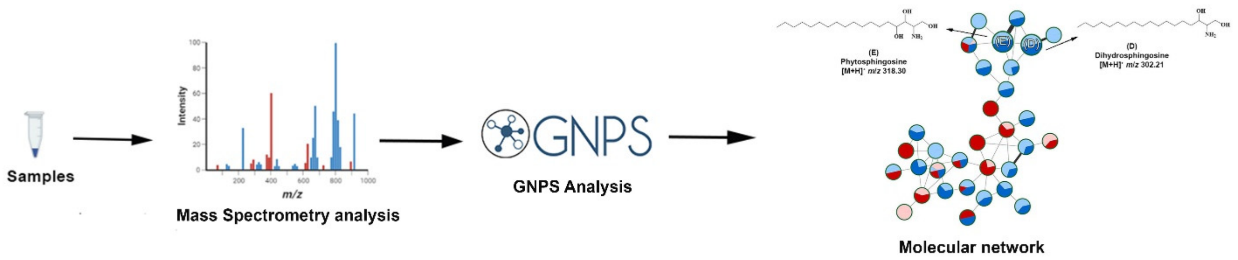

Figure S1. – Schematic representation of molecular network creation from tandem mass spectra data.

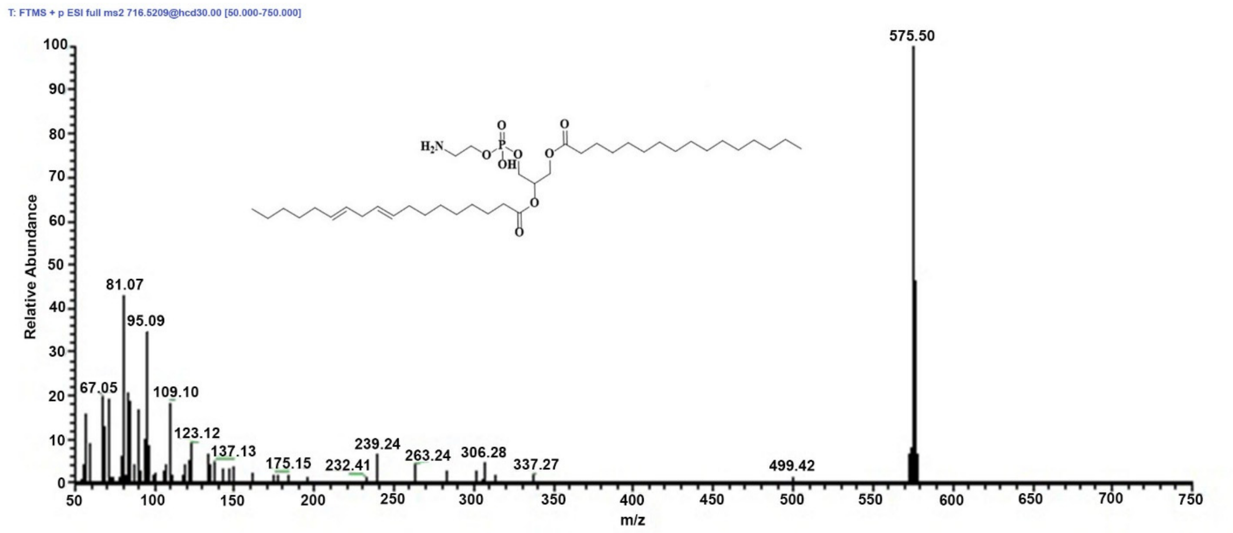

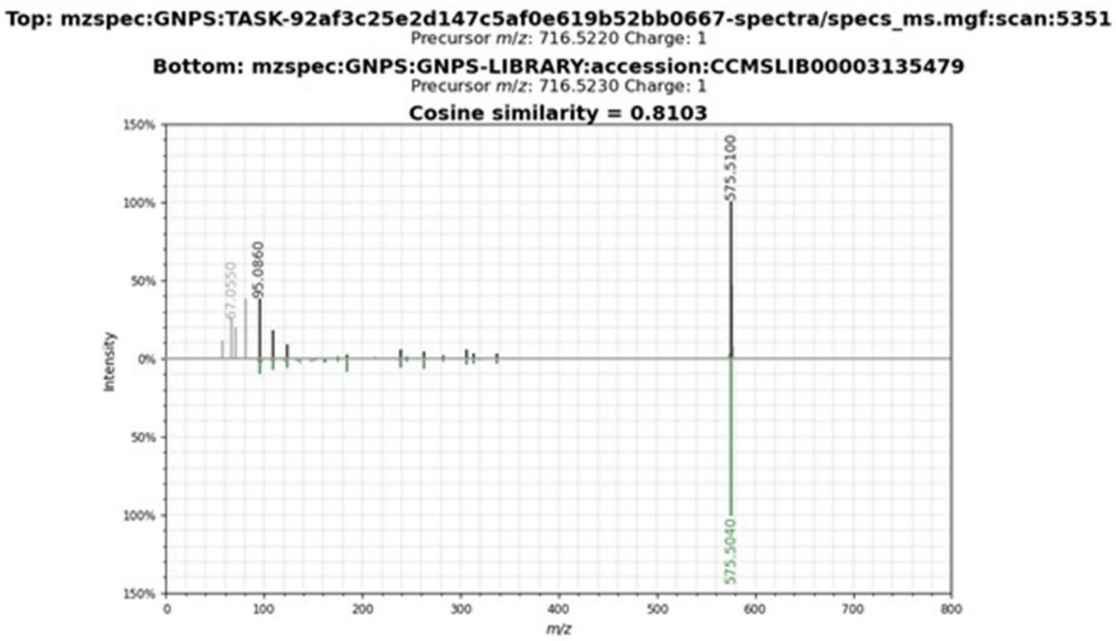

Figure S2. - The MS/MS fragmentation of 1-palmitoyl-2-linoleoyl-glycero-3-phosphoethanolamine and MS/MS match between GNPS.

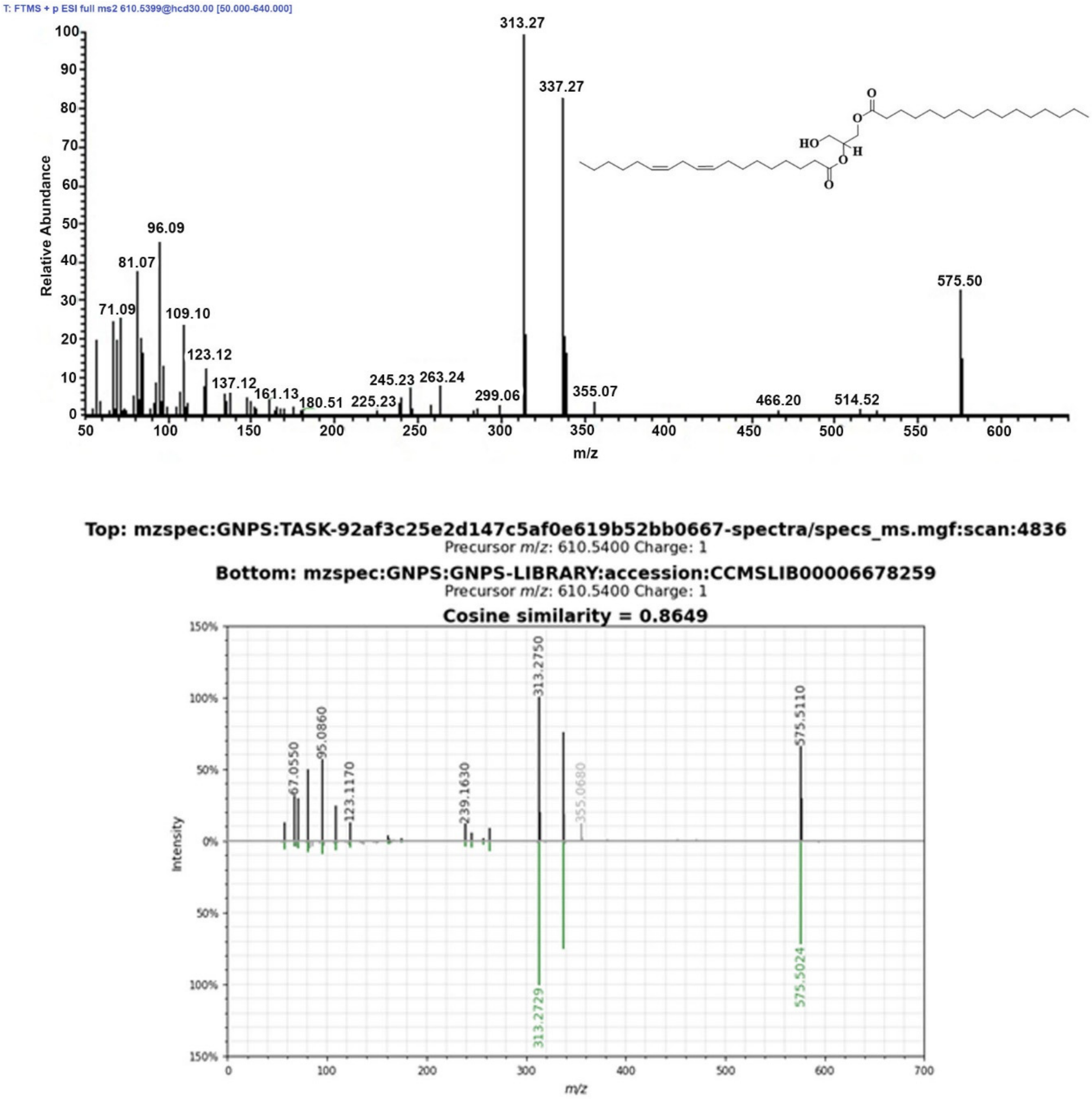

Figure S3. - The MS/MS fragmentation of 1-palmitoyl-2-linoleoyl-glycerol and MS/MS match between GNPS.

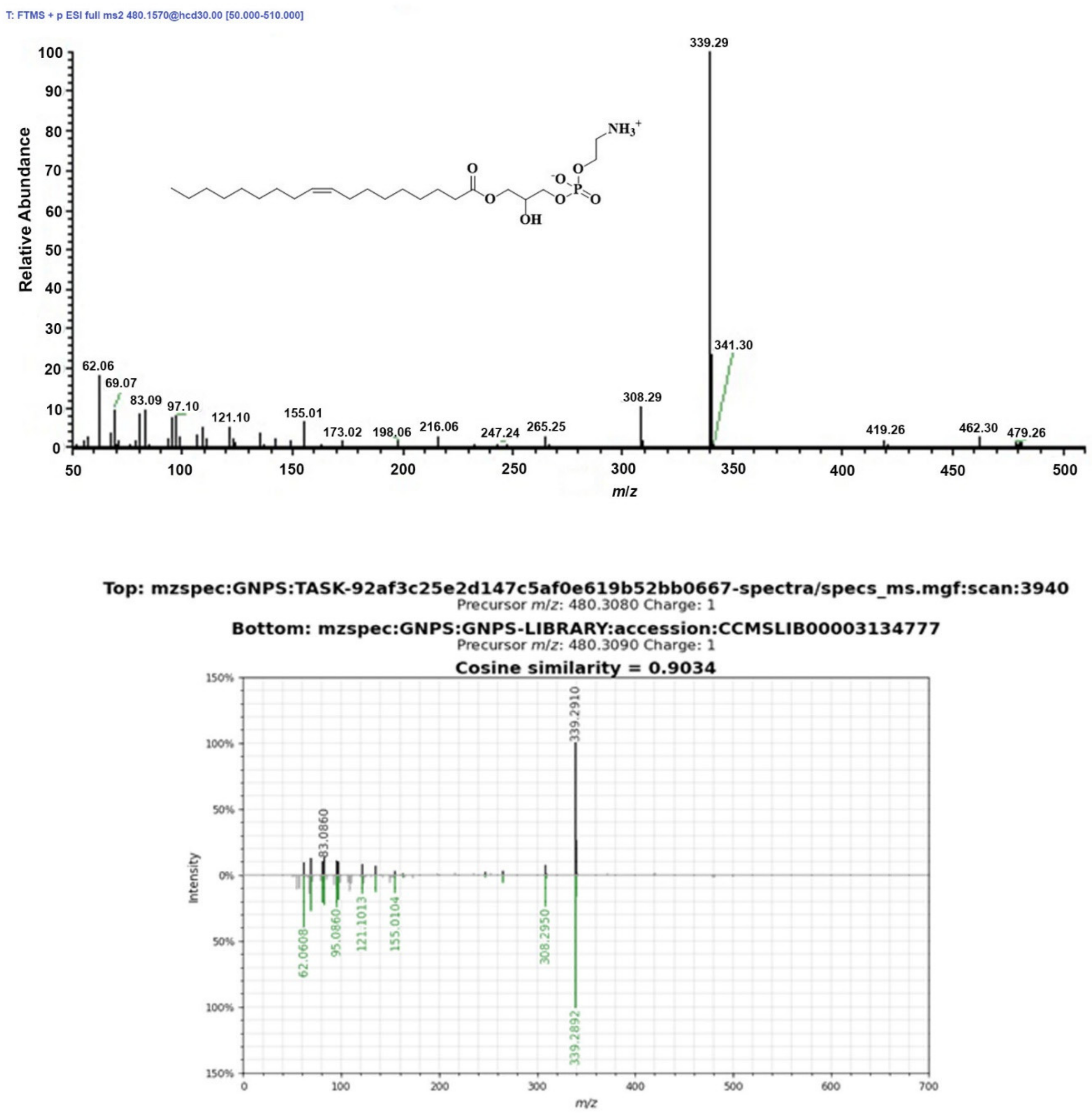

Figure S4. - The MS/MS fragmentation of 1-oleoyl-glycero-3-phosphoethanolamine and MS/MS match between GNPS database,

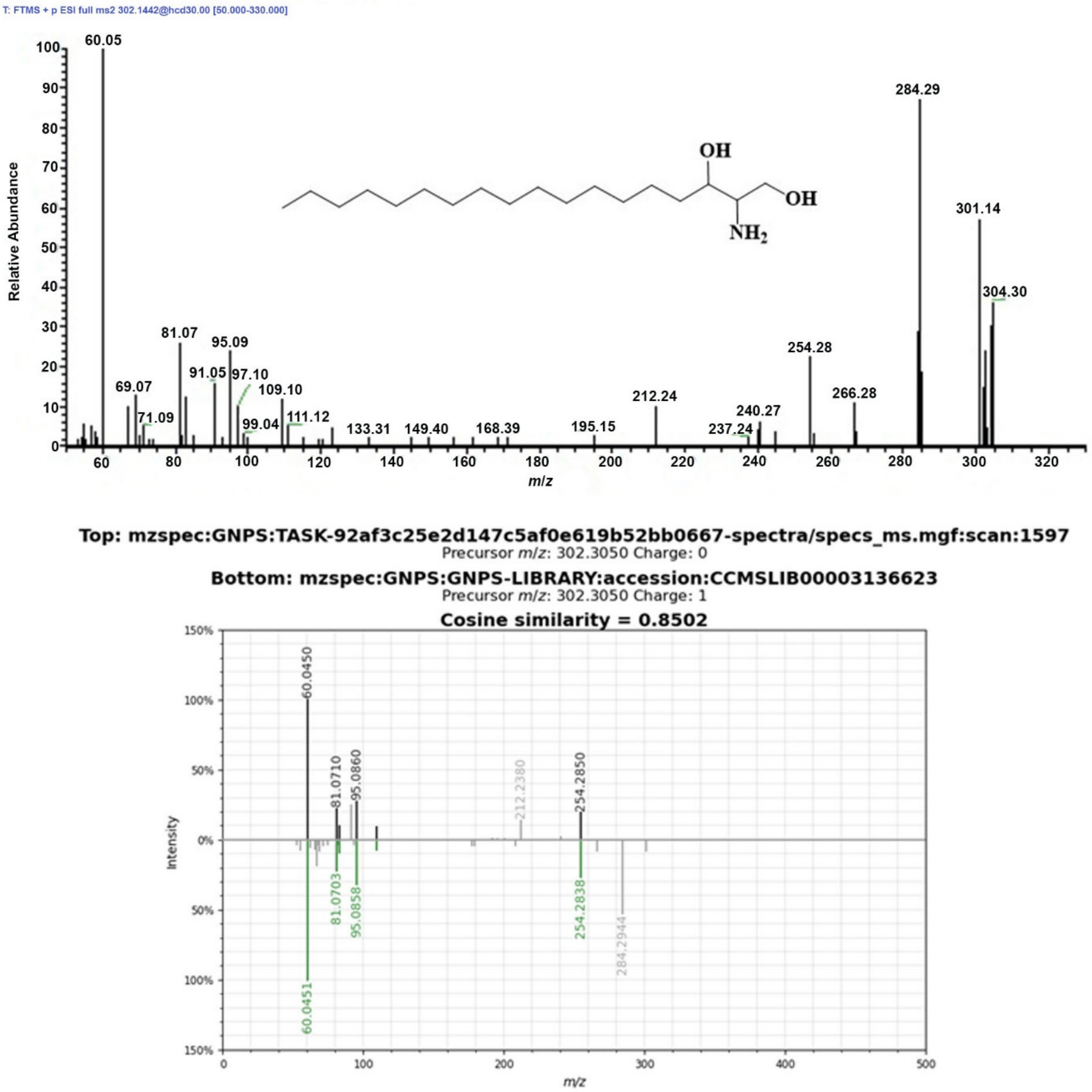

Figure S5. - The MS/MS fragmentation of dihydrosphingosine and MS/MS match between GNPS database.

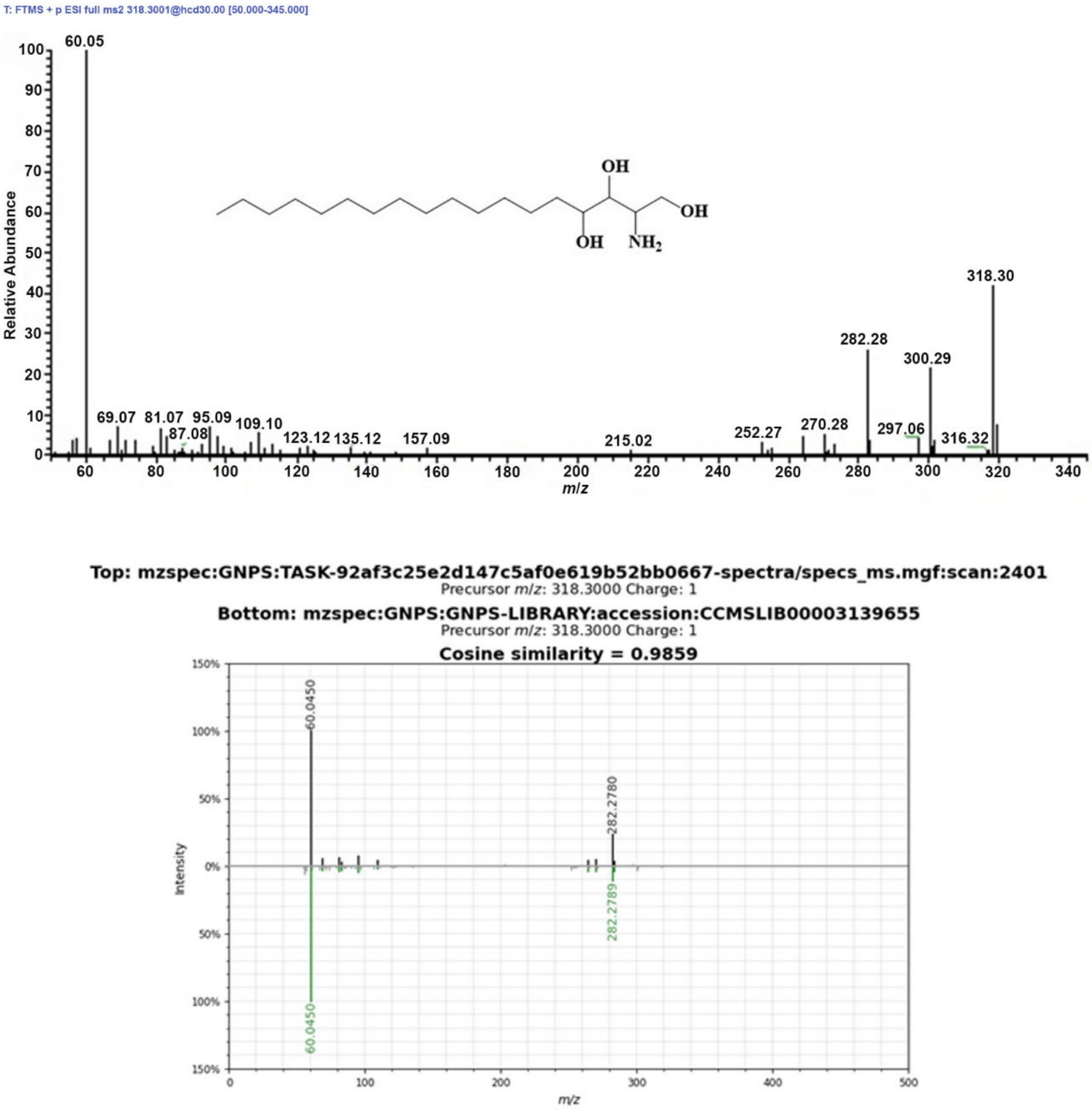

Figure S6. - The MS/MS fragmentation of phytosphingosine and MS/MS match between GNPS database.

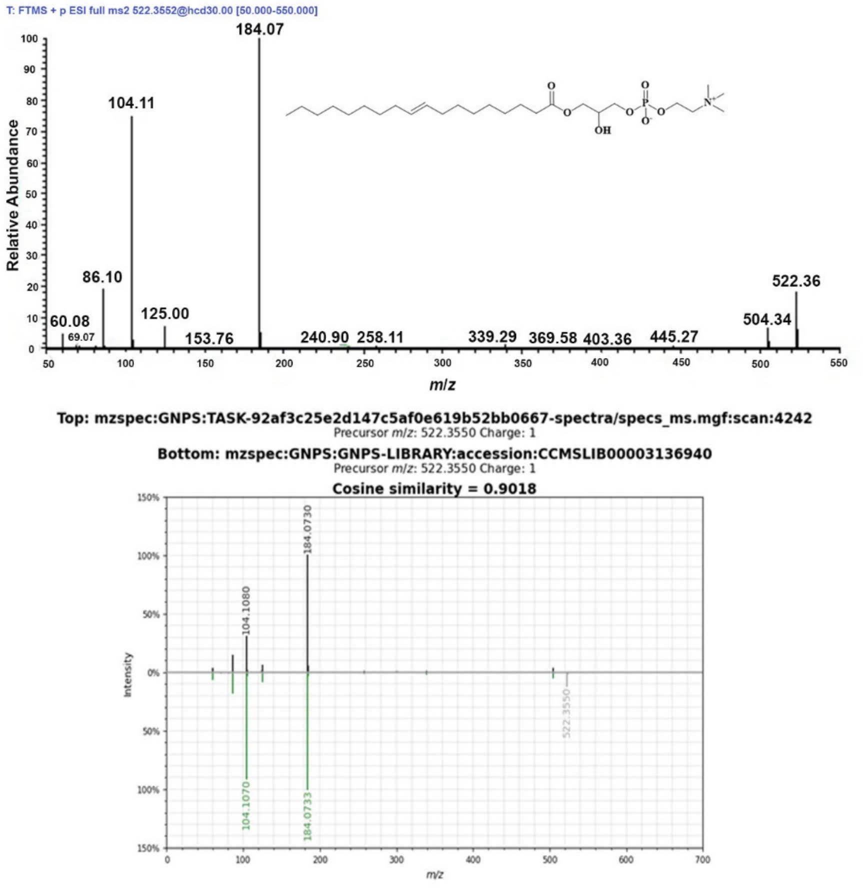

Figure S7. - The MS/MS fragmentation of 1-oleoyl-glycero-3-phosphocholine and MS/MS match between GNPS database.
